# Causal associations between kidney function and aortic valve stenosis: a bidirectional Mendelian randomization analysis

**DOI:** 10.1080/0886022X.2024.2417742

**Published:** 2024-10-23

**Authors:** Wanqian Pan, Le Zhou, Rui Han, Xiaojiao Du, Weixiang Chen, Tingbo Jiang

**Affiliations:** a Department of Cardiology, The First Affiliated Hospital of Soochow University, Suzhou, Jiangsu, China; b Department of Radiology, The First Affiliated Hospital of Soochow University, Suzhou City, Jiangsu Province, China

**Keywords:** Aortic valve stenosis, chronic kidney disease, estimated glomerular filtration rate, genome-wide association studies, kidney function, Mendelian randomization

## Abstract

**Background:**

Aortic valve stenosis (AVS) is currently the most common heart valve disease. The results of observational studies on the incidence of AVS in the renal dysfunction population are contradictory due to the short follow-up period and different diagnostic criteria, etc. This study aimed to explore the causal relationship between kidney function and AVS using Mendelian randomization (MR) analysis.

**Methods:**

We acquired summary statistics of estimated glomerular filtration rate (eGFR) and chronic kidney disease (CKD) from the CKDGen Consortium and a study on AVS from the FinnGen biobank. Univariate and multivariable MR analyses were conducted to evaluate the causal associations. The MR-Egger intercept and MR-PRESSO Global test were applied to assess the pleiotropic effects. The heterogeneity of MR results was tested by Cochran’s Q statistic. Moreover, the Bonferroni and FDR corrections were performed for multiple tests.

**Results:**

Genetically predicted decreased eGFR may be associated with a raised risk of AVS (OR = 0.045, *p* = 1.317e-04 by IVW; OR = 0.002, *p* = 0.004 by MR-Egger, OR = 0.091, *p* = 0.057 by WM). The causal association still established after multiple comparisons. Quality control analyses indicated the absence of heterogeneity and pleiotropy in our MR research. In addition, the causality of eGFR and AVS remained significant in multivariable MR analysis after adjusting BMI, hypertension, T2DM, LDL-C, and smoking.

**Conclusion:**

Our MR study discovered that reduced eGFR may be a causative risk factor for AVS. In addition, the evidence did not support a significant causal association of AVS on kidney function.

## Graphical Abstract

The present study utilized bidirectional Mendelian randomization analysis to investigate the causal association between kidney function and aortic valve stenosis. The reduced estimated glomerular filtration rate may be associated with an increased risk of aortic valve stenosis. No significant causal relationship was found in reverse MR analysis.

**Figure:**
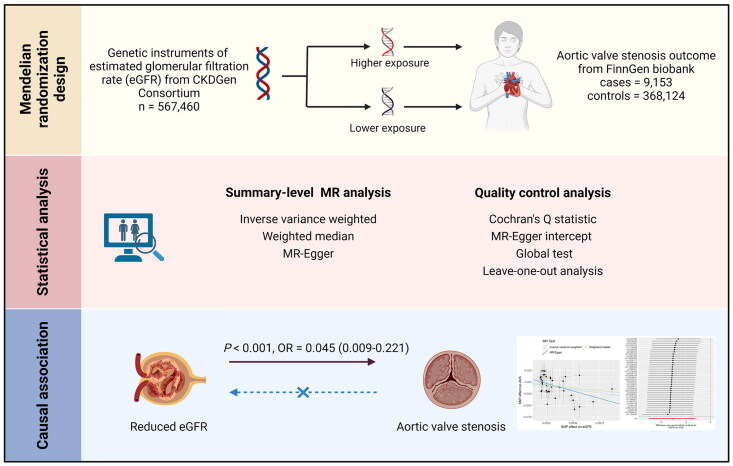

## Introduction

Aortic valve stenosis (AVS) is distinguished by the gradual calcification of the aortic valve leaflets, ranking as the most common valvular heart disease [1]. It confers significant mortality after clinical manifestation due to the obstruction of the left ventricular outflow tract [2,3]. In the United States of America, AVS accounts for 45% of all heart valve disease deaths as the potential cause of death [4]. Nevertheless, pharmacological therapies of AVS remain restricted [5]. Surgical aortic valve replacement has been recognized as the gold standard for the treatment of AVS for decades. Ultimately, many patients will require an intervention [6].

A better understanding of underlying mechanisms and risk factors is necessary for effective primary and secondary prevention of AVS [7]. Patients with impaired kidney function have a high prevalence of dyslipidemia [8], volume overload [9], and bone mineral metabolism [10], which are well-identified contributors to valve calcification [11]. In a prospective echocardiographic study, Maher ER and his colleagues reported that dialysis patients had an increased incidence of premature valve calcification and AVS [12]. However, Ix JH et al. observed only a modest association (*p* = 0.06) between impaired renal function and aortic valve calcification by cross-sectional study [13]. These conflicting outcomes may stem from low case numbers and confounding factor bias, etc.

Mendelian randomization (MR) uses genetic variants as instrumental variables to mimic the randomization process that underpins the causal associations of the exposure on an outcome overcoming the problems of reverse causality and confounding inherent in observational studies [14]. Thus, the MR approach demonstrates conceptual similarities to a randomized controlled trial, yet it is more extensively utilized and cost-effective. In this study, a bidirectional MR analysis was conducted to elucidate the causal associations between kidney function and the risk of AVS.

## Method

### Study design

**Figure 1.**
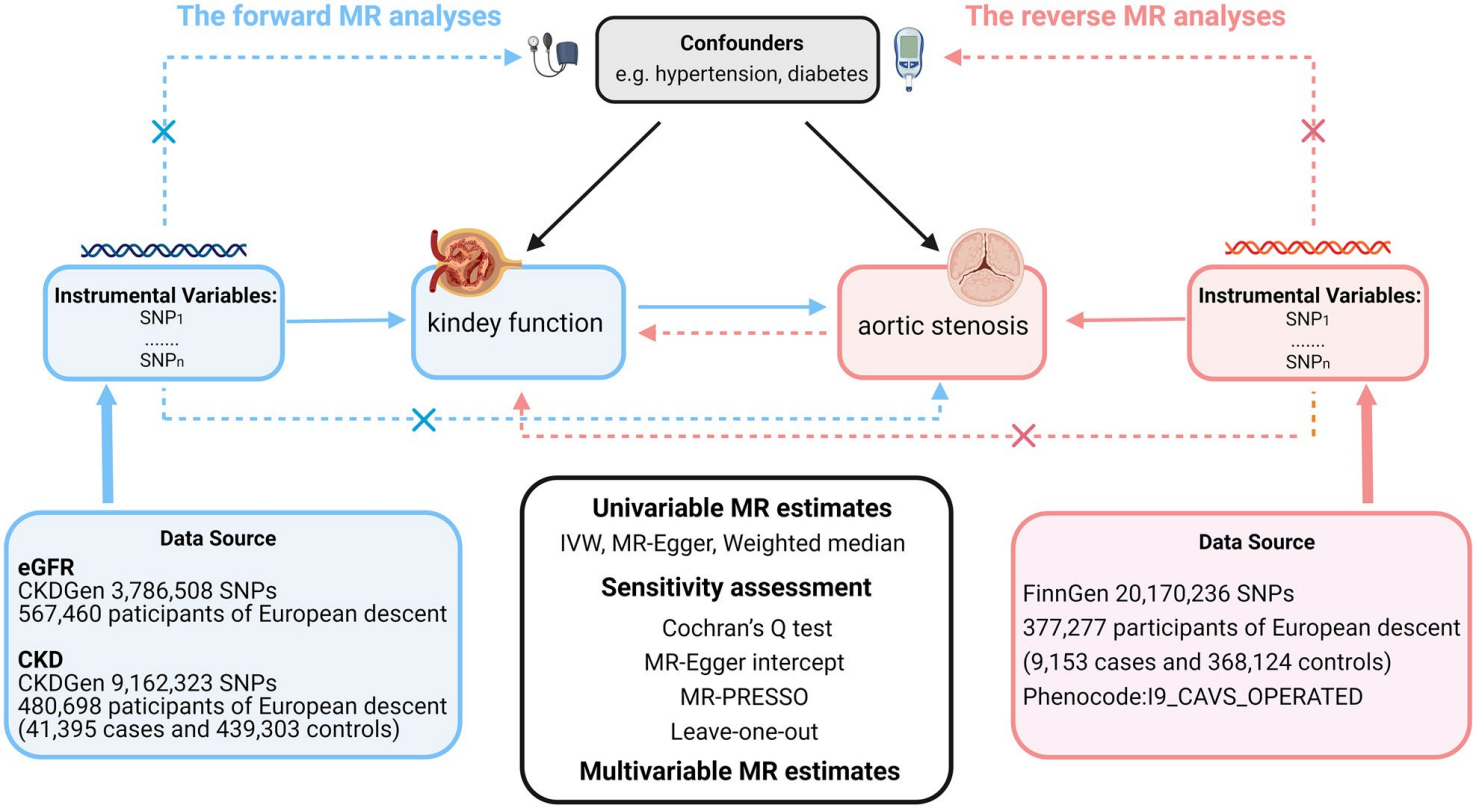
Study overview. This bidirectional MR analysis aims to assess the causal relationship between kidney function and AVS.

MR studies should satisfy the three main assumptions. First, instrumental variables should be reliably correlated with the exposure variables (the relevance assumption). Second, instrumental variables do not influence the outcome through confounders (the independence assumption). Third, instrumental variables are not directly related to the outcome (the exclusion restriction assumption). Figure 1 presents a summary of the principles, design, and procedure of this study.

### Data source

The CKDGen genome-wide association studies (GWAS) meta-analysis data was utilized to develop the genetic instruments for serum creatinine-based estimated glomerular filtration rate (eGFR) and chronic kidney disease (CKD). The summary genetic statistics for kidney function were derived from a 2019 study conducted by Wuttke M et al. [15]. The GWAS meta-analysis of eGFR encompassed a cohort of 567,460 individuals of European ancestry across 54 cohorts. The calculation of GFR involved the application of the Chronic Kidney Disease Epidemiology Collaboration equation for adults (age > 18 years) and the Schwartz formula for those aged 18 years or younger. The data of CKD (defined as eGFR < 60 mL/min/1.73m^2^) included 23 cohorts of European ancestry containing 41,395 cases and 439,303 controls.

The summary statistics of AVS were extracted from the GWAS in the FinnGen Project Database, consisting of 377,277 participants (9,153 cases and 368,124 controls) of European ancestry. AVS was diagnosed on the basis of ICD codes (I35.0 and I35.2), which correspond to nonrheumatic aortic stenosis (Table S1).

### Selection of genetic instrumental variants

To meet the three key assumptions, we first screened for single-nucleotide polymorphisms (SNPs) associated with exposure and eliminated linkage disequilibrium with *p* < 5 × 10^−8^, r^2^ < 0.001, and kb > 10,000 [16]. Secondly, we searched the traits linked to each SNP using the PhenoScannerV2 database. The instrumental variables surrogating the confounders (e.g. body mass index, hypertension, diabetes) and outcomes were deleted to satisfy the second and third assumptions. Thirdly, harmonization of instrumental variables for exposure and outcome to remove palindromic SNPs. Fourthly, the MR-PRESSO method was applied to test and remove all outlier SNPs. Finally, we computed the proportion of trait variance explained by each SNP (R^2^) using the equation: R^2^ = [2 × (1 – MAF) × MAF × β^2^]/(SE^2^ × N), where SE and β are the standard error and β coefficient for effect size, MAF presents the minor allele frequency for each SNP, and N is the sample size. Then, we employed the *F*-statistics for SNPs to assess the strength of the instrumental variables, which is calculated as [(N - k − 1)/k] × [R^2^/(1 - R^2^)], where N denotes the sample size, k refers to the number of SNPs, and R^2^ is the proportion of inter-individual variance in phenotypic variations explained. *F*-statistics ≥ 10 are defined as strong genetic instruments for accounting for variation in phenotypes [17]. Only strong instrumental variables were included to avoid the risk of weak instrument bias. In this study, we respectively obtained 36 and six independent genetic SNPs associated with eGFR and CKD. Similarly, we selected seven and six genetic instrumental variables representing AVS for reverse MR analysis with eGFR and CKD (Tables S2–S5).

### MR analysis

After identifying the SNPs of exposure, we matched the same SNP and its corresponding statistical information in the GWAS of outcome. Based on the *P*-value, beta, standard error, alleles et al. TwoSampleMR R package was conducted to evaluate the causal associations between kidney function and AVS using three different approaches: inverse variance weighted (IVW) with fixed-effects model, MR-Egger, and weighted median (WM) [18]. By aggregating the Wald ratios of genetically causal effects for each instrumental variable, the IVW approach operates on the assumption that all SNPs are valid instrumental variants [19]. Thus, it is regarded as offering the most powerful and precise estimate, serving as the primary analysis [20]. When up to half of SNPs are considered unreliable instrumental variables, the WM approach provides a summed measure of the overall effect [21]. The MR-Egger approach permits the incorporation of directional genetic pleiotropy in the included SNPs [22]. In addition, we assessed statistical power using the mRnd power calculator, available at https://shiny.cnsgenomics.com/mRnd/

To reduce the rate of false positives caused by the stochastic effect, we performed Bonferroni and false discovery rate (FDR) correction methods to correct the results for multiple comparisons. The threshold of significance after Bonferroni-adjustment multiple testing was *p* < 0.025 (0.05/2 = 0.025). An FDR-adjusted *P*-value (PFDR) below 0.05 was used to identify significant causal associations.

Given the possible influence of confounding factors, multivariable MR analysis was performed to evaluate the direct effect of eGFR on AVS whilst adjusting for potential confounding factors [2], including body mass index (BMI), hypertension, type 2 diabetes mellitus (T2DM), low-density lipoprotein-cholesterol (LDL-C), and smoking (Table S1).

### Heterogeneity, pleiotropy, and sensitivity assessment

Heterogeneity among the causal estimates across all SNPs was measured by calculating Cochran’s Q statistic. I^2^ (%)-value was calculated by formula [Q-(K-1)]/Q to quantitatively assess the heterogeneity. K represents the count of SNPs [19]. The MR-Egger intercept and MR-PRESSO Global test methods were applied to assess the presence of horizontal pleiotropy. A *P*-value exceeding 0.05 suggests no pleiotropy [23]. Leave-one-out sensitivity tests were used to assess if a unique SNP dominates the assessment of causality [24].

### Statistical analysis

The ‘TwoSampleMR’, ‘LDlinkR’, and ‘forestplot’ packages were employed for causality estimation in R software 4.3.1.

## Results

### Causal relationship estimates for kidney function on AVS

**Figure 2.**
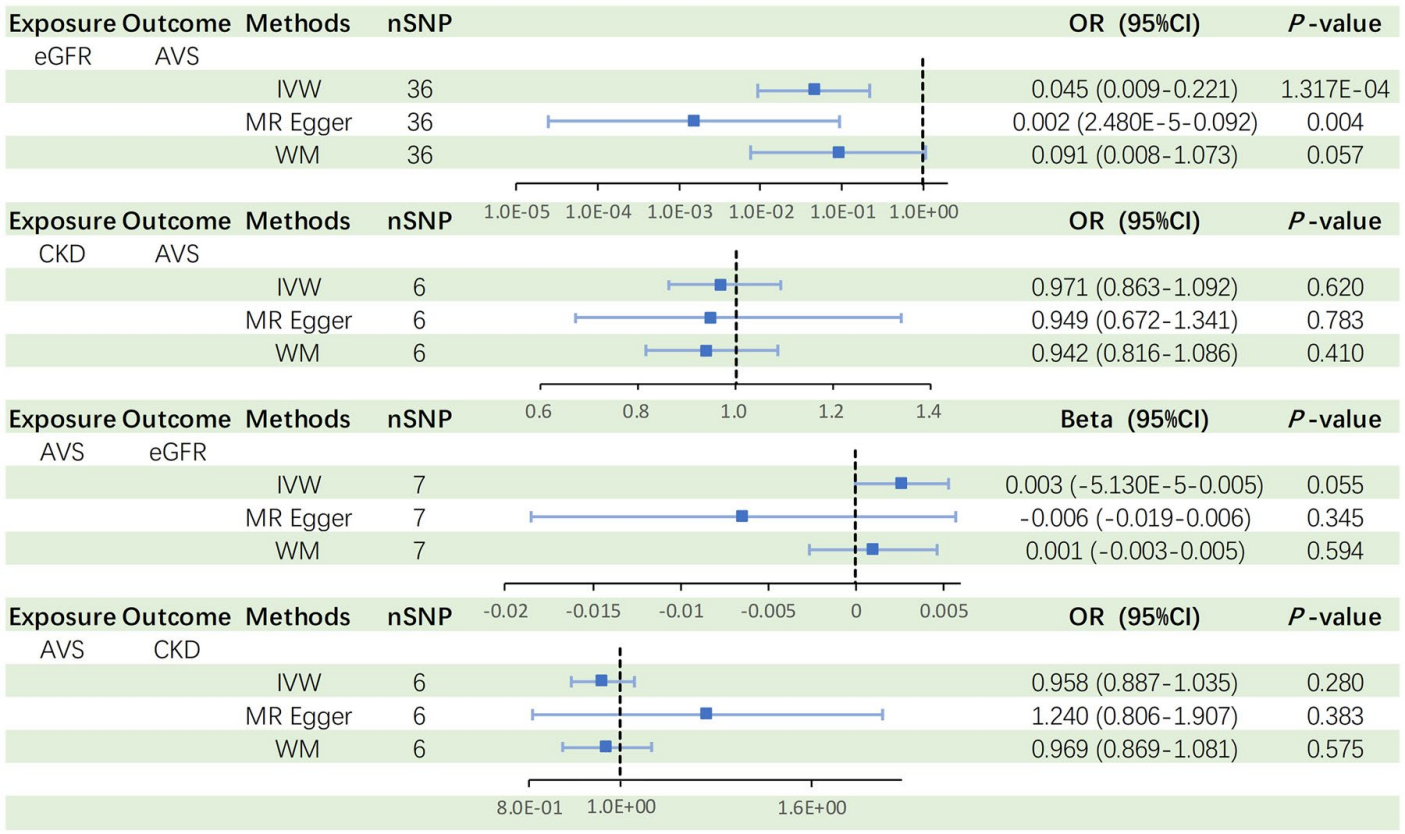
Summary-level MR analysis results. ORs derived from the three MR methods are visualized in the Forest plot. *P*-values and 95CI% specific values are listed on the right side.

**Figure 3.**
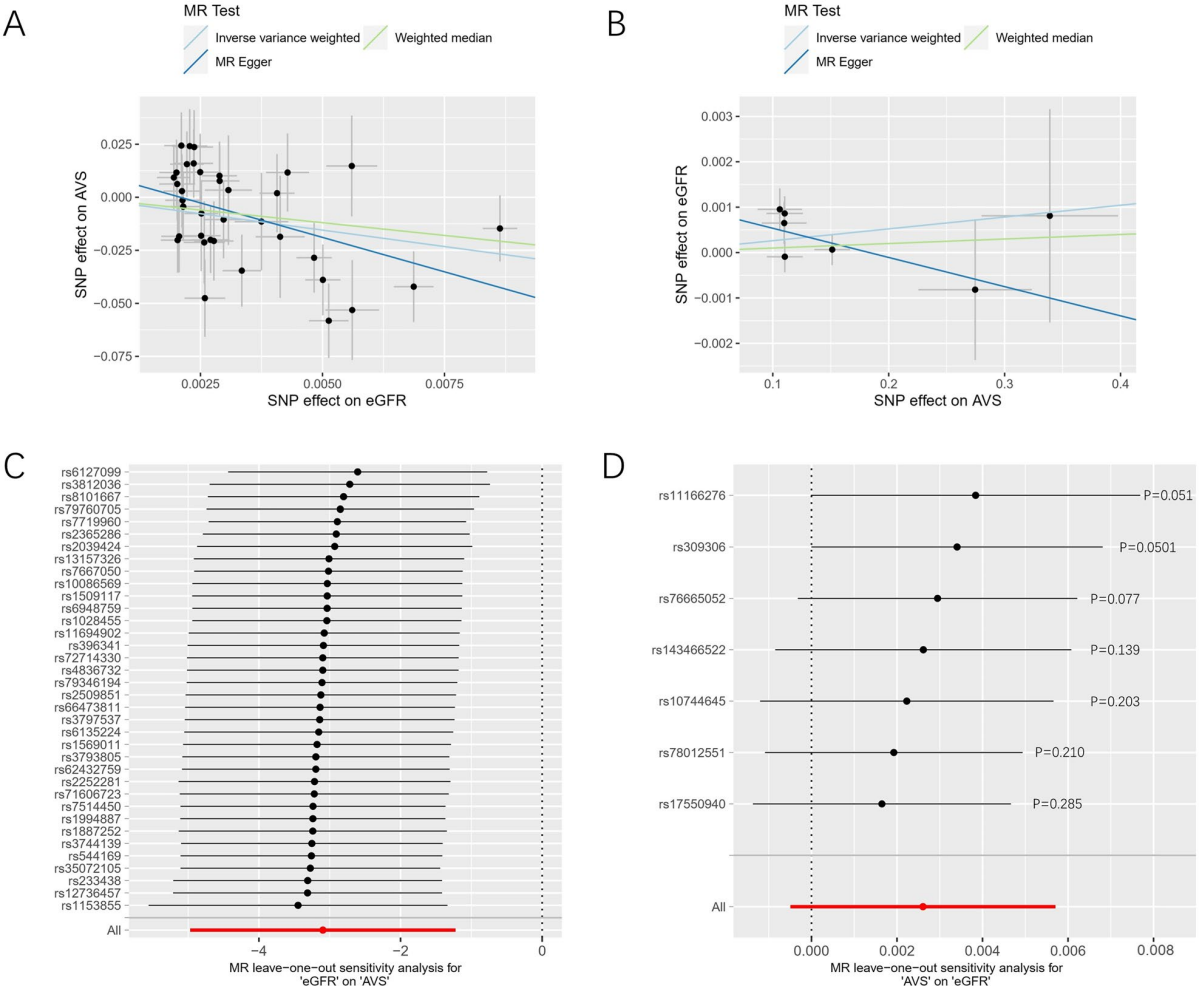
Scatter plots of causal associations and leave-one-out plots of the sensitivity analysis. (A) Scatter plot for estimating the change in eGFR on the risk of AVS. (B) Scatter plot for estimating the risk of AVS on the change in eGFR. (C) Leave-one-out analysis for eGFR on AVS. (D) leave-one-out analysis for AVS on eGFR.

As summarized in Figure 2, the *P*-values derived from the IVW and MR Egger methods were 1.317e-04 (95% CI: 0.009-0.221, OR = 0.045) and 0.004 (95% CI: 2.480E-05-0.092, OR = 0.002), respectively. The association was still established after applying the Bonferroni adjusted significance level. In addition, the *P*_FDR_ for IVW and MR-Egger methods are respectively 2.624e-04 and 0.008, which demonstrated that the causality between eGFR and AVS remained significant after multiple tests. The WM method results exhibited a consistent trend, although it did not reach statistical significance (*p* = 0.057). The scatter plots of the three methods are shown in Figure 3A. All the trend lines suggested that genetically predicted decreased eGFR was associated with increased incidence of AVS. Figure S1 exhibits the forest plot of individual SNP effect of eGFR on AVS. However, we did not observe an association between CKD and AVS (*p* = 0.620, 95%CI: 0.863-1.092).

### Multivariable MR analysis

Considering the potential effects of various risk factors, we performed the multivariable MR analysis to accurately assess the direct effect of eGFR on AVS **(**Table 1**)**. The multivariable MR results show that the causal effects of eGFR on AVS remained significant, after adjusting for BMI, hypertension, T2DM, LDL-C, and smoking.

**Table 1.** The results of multivariable MR analysis.

Exposure	nSNP	OR (95 % CI)	*P*-value
eGFR + BMI	449	0.082 (0.021-0.315)	2.656E-04
eGFR + Hypertension	206	0.290 (0.095-0.885)	0.030
eGFR + T2DM	211	0.277 (0.095-0.807)	0.019
eGFR + LDL-C	323	0.240 (0.067-0.852)	0.027
eGFR + smoking	201	0.359 (0.131-0.986)	0.047

### Quality control assessment of forward MR analysis

We employed Cochran’s Q statistic for the analysis of heterogeneity. As shown in Table 2, there was no significant heterogeneity among the variant-specific effects (eGFR: I^2^ = 0.282, *p* = 0.061; CKD: I^2^ = 0.244, *p* = 0.251). Subsequently, we assessed pleiotropy through the MR-Egger intercept and Global test approaches. The *P*-values from both approaches were greater than 0.05, signifying the absence of pleiotropic variants within the selected SNPs. Figure 3C illustrates the leave-one-out analysis depicting the impact of the individual eGFR-SNPs on the overall causal estimate of the risk of AVS. These findings suggested that our MR results were robust and steady, with no noticeable alterations in total effect estimates detected when any one SNP was removed and returned. Ultimately, we evaluated the statistical power of the MR analysis, yielding a result of 0.99. This finding signified a strong capability to detect a significant causal association of eGFR changes with AVS.

**Table 2.** Heterogeneity, pleiotropy, and statistical power evaluations of MR analyses.

Exposure	Outcome	Cochran’s Q statistic	*P*-value for Cochran’s Q	Egger intercept	*P*-value for Egger intercept	*P*-value for Global Test	Power of MR	*F* statistic
eGFR	AVS	48.751	0.061	0.014	0.080	0.052	0.990	34
CKD	AVS	6.613	0.251	0.003	0.896	0.371	0.050	18
AVS	eGFR	8.158	0.227	0.001	0.193	0.263	0.050	16
AVS	CKD	10.035	0.074	−0.035	0.294	0.140	0.050	15

### Causal relationship estimates for AVS on kidney function

We performed reverse directional MR analyses to explore the causal impact of AVS on kidney function (Tables S4 and S5). As shown in Figure 2, the MR results revealed a non-significant association between genetically predicted AVS and eGFR (95% CI: 1.000-1.005, *p* = 0.055 by IVW, 95% CI: 0.982-1.006, *p* = 0.345 by MR-Egger, 95% CI: 0.997-1.005, *p* = 0.594 by WM). After conducting the Cochran’s Q test (*Q* = 8.158, I^2^ = 0.264, *p* = 0.227), Global test (*p* = 0.263), and MR-Egger intercept (*p* = 0.193) methods, the analysis demonstrated the absence of both heterogeneity and pleiotropy in the seven AVS-SNPs (Table 2). The leave-one-out plot illustrates that no individual SNP significantly influenced the causal relationship between AVS and eGFR (Figure 3D).

Similarly, there was no significant causal association of AVS on the odds of CKD (Figure 2). Forest and scatter plots depicting the causal association of AVS with CKD are presented in Figures S1 and S2.

## Discussion

We evaluated the causal relationship between kidney function and the risk of AVS based on the use of large available GWAS statistics. This study revealed a significant association, wherein lower genetically predicted eGFR may be linked to an increased incidence of AVS. However, we did not find evidence to support the causality of AVS on kidney function. The results suggested that preventing a decline in eGFR may alleviate the public health burden of AVS.

The WM method requires more than 50% of the instrumental variables to be valid, and it is more sensitive to heterogeneity and outliers [18,21]. Based on the predefined decision tree, WM is recommended as the primary method for MR analysis when heterogeneity is present (Figure S3) [25,26]. The present MR study did not find significant heterogeneity and pleiotropy. Therefore, the IVW method should be used as the primary outcome. The MR-Egger method further supported our conclusion. Although the WM method did not demonstrate a statistical difference, it was in the same direction as the other methods [27], demonstrating that the included SNPs were stable. As a result, we could still consider that there may be a causal effect between eGFR and AVS. Moreover, we conducted Bonferroni and FDR corrections to reduce type I error. The results demonstrated that the causal relationship between eGFR and AVS still held after multiple tests.

Although MR analysis has shown that reduced eGFR may be associated with an increased risk of AVS, we did not observe a causal effect between CKD and AVS. We speculate the reasons are as follows. Firstly, the eGFR is a continuous variable, whereas CKD is a dichotomous variable, which is defined according to eGFR < 60 mL/min/1.73m^2^. In this study, the GWAS meta-analysis of eGFR encompassed a cohort of 567,460 individuals, whereas the summary statistics for CKD included 41,395 cases and 439,303 controls. Detecting the effect of exposure factors on outcomes becomes more difficult when the proportion of cases is small. Statistical significance may not be reached, as the uncertainty of effect estimates increases. This means that even if there is a true causal effect, the study may not be able to detect it. Secondly, smaller decreases in eGFR may be sufficient to trigger pathological changes in the aortic valve, but diagnostic criteria for CKD require a significant decrease in eGFR. As a result, it fails to capture the causal effect. Finally, the diagnosis of CKD should also reference renal structure and other indicators of renal function (e.g. urine sediment, urinary albumin, etc.) [28]. Limited by the database, the GWAS statistics of CKD that we utilized were based on eGFR diagnosis, which does not fully represent the instrumental variables of CKD. Therefore, further studies are needed to investigate the causal effect between CKD and AVS.

Several observational studies have demonstrated that AVS incidence increases in a dose-dependent manner as renal function declines [29–32]. Based on clinical diagnostic codes from the Stockholm CREAtinine Measurements project, Vavilis G et al. found that slight renal dysfunction with eGFR 60 to 90 mL/min/1.73 m^2^ was related to higher hazards of AVS (hazard ratio 1.14) [29]. Furthermore, Samad Z et al. reported that the prevalence of at least mild AVS was 9.5% in patients with CKD *versus* 3.5% in the non-CKD group by utilizing echocardiogram [32]. The process of aortic valve calcification occurs at an early age among renal dysfunctional patients and progresses rapidly [30,33].

The study indicated that hemodynamic disturbance and endothelial injury by hypertension and hemodialysis are involved in the pathophysiological mechanisms of valvular lesions [34]. What’s more, oxidized low-density lipoprotein and lipoprotein (a)-mediated inflammation contributes to the development of AVS in the uremic milieu [35]. Receptor activator of nuclear factor κ-B (RANKL) activation induced by bone mineral disorder will promote calcification and osteogenic transdifferentiation of vascular smooth muscle cells [36]. Lerman DA et al. reported that RANKL antagonists can be used as prophylactic agents for aortic valve calcification through assessing the calcification degree of porcine valvular interstitial cells [37]. Thus, targeted therapy may potentially slow the development of AVS in patients with impaired renal function. However, the efficacy of these antagonist drugs needs to be further investigated.

However, in a large computer tomography-based observational study, impaired kidney function failed to associate with the presence of AVS [13]. The Framingham Offspring Study also did not observe an association between CKD and aortic annular calcification or aortic sclerosis by using echocardiography [38]. Different assessment criteria and insufficient follow-up time would lead to these contradictory results. In addition, a causal effect could not be confirmed from previous observational studies due to the diagnostic delay and common risk factors of both renal impairment and AVS [39].

Based on the principle of natural random assignment of alleles inherited by the offspring from the parents during the formation of the zygote, MR analyses can draw reliable causal inferences without potential confounding bias and reverse causality [40]. Our dataset was sourced from the CKDGen Consortium and a comprehensive AVS-GWAS dataset, ensuring no overlap in samples. Compared to previous studies, we screened instrumental variables to avoid confounding bias by searching in the PhenoScannerV2 database [29,41]. We excluded SNPs associated with confounders and selected strong instruments for the analyses. We conducted three different analytical approaches to efficiently estimate MR causal effect sizes. In addition, we conducted multiple testing to decrease the possibility of false positives. Multivariable MR analyses were performed to reduce the potential effects of various risk factors. Assessments of sensitivity, heterogeneity, and pleiotropy demonstrated that our results were robust and reliable.

The implications of our study for clinical practice are summarized below. First, as impaired renal function may be a causal risk factor of AVS, early and regular evaluation of aortic valve changes is essential in patients with low eGFR levels. Second, in patients with established renal insufficiency, early screening and diagnosis of AVS may help treat timely and prevent progression to possibly reduce mortality of valvular disease. Finally, effective management of renal insufficiency and its comorbidities may be potentially beneficial in mitigating the incidence of AVS. We still need to conduct large-scale randomized controlled trials to determine its effectiveness, including proprotein convertase subtilisin/kexin type 9 inhibitors and SNF472 [42,43].

Our study has some limitations that need to be emphasized. Firstly, the current study was centered on European ethnicity, which limited the application of causal inferences to other populations. Second, the magnitude of the impact of clinical interventions cannot be determined by MR analysis. Third, owing to the unavailability of publicly available data, our study could not assess the development and severity of AVS. Finally, the complex mechanism of causal relationship between renal dysfunction and AVS should be validated in future biological experiments.

## Conclusion

In conclusion, our MR analyses showed that genetically determined decreased eGFR may raise the risk of AVS. Contrarily, the reverse directional MR analysis did not substantiate a causal association of AVS risk on kidney function. This study may guide the management of patients with renal dysfunction, which may contribute to curbing the global epidemic of AVS.

## Supplementary Material

Figure1.png

Supplementary Materials.docx

Figure2.tif

Figure3.tif

Graphical abstract.tif

## Data Availability

There is no use of raw, unprocessed data in the current study. The GWAS summary statistics underlying the findings of this study are publicly accessible in the CKDGen Consortium (http://ckdgen.imbi.uni-freiburg.de) and FinneGen biobank (phenocode: I9_CAVS_OPERATED, https://www.finngen.fi/en/access_results).

## References

[1] Moncla L-HM, Briend M, Bossé Y, et al. Calcific aortic valve disease: mechanisms, prevention and treatment. Nat Rev Cardiol. 2023;20(8):546–559. doi: 10.1038/s41569-023-00845-7. 36829083

[2] Boskovski MT, Gleason TG. Current therapeutic options in aortic stenosis. Circ Res. 2021;128(9):1398–1417. doi: 10.1161/CIRCRESAHA.121.318040. 33914604

[3] Chan KL. Is aortic stenosis a preventable disease? J Am Coll Cardiol. 2003;42(4):593–599. doi: 10.1016/s0735-1097(03)00786-1. 12932587

[4] Coffey S, Cairns BJ, Iung B. The modern epidemiology of heart valve disease. Heart. 2016;102(1):75–85. doi: 10.1136/heartjnl-2014-307020. 26541169

[5] Baumgartner H, Falk V, Bax JJ, et al. 2017 ESC/EACTS guidelines for the management of valvular heart disease. Eur Heart J. 2017;38(36):2739–2791. doi: 10.1093/eurheartj/ehx391. 28886619

[6] Joseph J, Naqvi SY, Giri J, et al. Aortic stenosis: pathophysiology, diagnosis, and therapy. Am J Med. 2017;130(3):253–263. doi: 10.1016/j.amjmed.2016.10.005. 27810479

[7] Aluru JS, Barsouk A, Saginala K, et al. Valvular heart disease epidemiology. Med Sci (Basel). 2022;10(2):32. doi: 10.3390/medsci10020032. PMC9228968 35736352

[8] Suh SH, Kim SW. Dyslipidemia in patients with chronic kidney disease: an updated overview. Diabetes Metab J. 2023;47(5):612–629. doi: 10.4093/dmj.2023.0067. 37482655 PMC10555535

[9] Matsushita K, Ballew SH, Wang AY-M, et al. Epidemiology and risk of cardiovascular disease in populations with chronic kidney disease. Nat Rev Nephrol. 2022;18(11):696–707. doi: 10.1038/s41581-022-00616-6. 36104509

[10] Williams ME. Chronic kidney disease/bone and mineral metabolism: the imperfect storm. Semin Nephrol. 2009;29(2):97–104. doi: 10.1016/j.semnephrol.2009.01.002. 19371800

[11] Hutcheson JD, Goettsch C. Cardiovascular calcification heterogeneity in chronic kidney disease. Circ Res. 2023;132(8):993–1012. doi: 10.1161/CIRCRESAHA.123.321760. 37053279 PMC10097496

[12] Maher ER, Young G, Smyth-Walsh B, et al. Aortic and mitral valve calcification in patients with end-stage renal disease. Lancet. 1987;2(8564):875–877. doi: 10.1016/s0140-6736(87)91370-5. 2889080

[13] Ix JH, Shlipak MG, Katz R, et al. Kidney function and aortic valve and mitral annular calcification in the Multi-Ethnic Study of Atherosclerosis (MESA). Am J Kidney Dis. 2007;50(3):412–420. doi: 10.1053/j.ajkd.2007.05.020. 17720520

[14] Davey Smith G, Hemani G. Mendelian randomization: genetic anchors for causal inference in epidemiological studies. Hum Mol Genet. 2014;23(R1):R89–98. doi: 10.1093/hmg/ddu328. 25064373 PMC4170722

[15] Wuttke M, Li Y, Li M, et al. A catalog of genetic loci associated with kidney function from analyses of a million individuals. Nat Genet. 2019;51(6):957–972. doi: 10.1038/s41588-019-0407-x. 31152163 PMC6698888

[16] Chen X, Kong J, Pan J, et al. Kidney damage causally affects the brain cortical structure: a Mendelian randomization study. EBioMedicine. 2021;72:103592. doi: 10.1016/j.ebiom.2021.103592. 34619639 PMC8498227

[17] Burgess S, Thompson SG. Avoiding bias from weak instruments in Mendelian randomization studies. Int J Epidemiol. 2011;40(3):755–764. doi: 10.1093/ije/dyr036. 21414999

[18] Burgess S, Davey Smith G, Davies NM, et al. Guidelines for performing Mendelian randomization investigations: update for summer 2023. Wellcome Open Res. 2019;4:186. doi: 10.12688/wellcomeopenres.15555.1. 32760811 PMC7384151

[19] Burgess S, Dudbridge F, Thompson SG. Combining information on multiple instrumental variables in Mendelian randomization: comparison of allele score and summarized data methods. Stat Med. 2016;35(11):1880–1906. doi: 10.1002/sim.6835. 26661904 PMC4832315

[20] Marouli E, Del Greco MF, Astley CM, et al. Mendelian randomisation analyses find pulmonary factors mediate the effect of height on coronary artery disease. Commun Biol. 2019;2(1):119. doi: 10.1038/s42003-019-0361-2. 30937401 PMC6437163

[21] Bowden J, Davey Smith G, Haycock PC, et al. Consistent estimation in mendelian randomization with some invalid instruments using a weighted median estimator. Genet Epidemiol. 2016;40(4):304–314. doi: 10.1002/gepi.21965. 27061298 PMC4849733

[22] Burgess S, Thompson SG. Interpreting findings from Mendelian randomization using the MR-Egger method. Eur J Epidemiol. 2017;32(5):377–389. doi: 10.1007/s10654-017-0255-x. 28527048 PMC5506233

[23] Greco MF, et al. Detecting pleiotropy in Mendelian randomisation studies with summary data and a continuous outcome. Stat Med. 2015;34(21):2926–2940. 25950993 10.1002/sim.6522

[24] Verbanck M, Chen C-Y, Neale B, et al. Detection of widespread horizontal pleiotropy in causal relationships ­inferred from Mendelian randomization between complex traits and diseases. Nat Genet. 2018;50(5):693–698. doi: 10.1038/s41588-018-0099-7. 29686387 PMC6083837

[25] Jiang Q, Qin D, Yang L, et al. Causal effects of plasma lipids on the risk of atrial fibrillation: a multivariable mendelian randomization study. Nutr Metab Cardiovasc Dis. 2021;31(5):1569–1578. doi: 10.1016/j.numecd.2021.02.011. 33814236

[26] Guan B, Chen X-Q, Liu Y, et al. Causal effects of circulating vitamin levels on the risk of heart failure: a Mendelian randomization study. J Geriatr Cardiol. 2023;20(3):195–204. doi: 10.26599/1671-5411.2023.03.007. 37091260 PMC10114193

[27] Ji D, Chen W-Z, Zhang L, et al. Gut microbiota, circulating cytokines and dementia: a Mendelian randomization study. J Neuroinflammation. 2024;21(1):2. doi: 10.1186/s12974-023-02999-0. 38178103 PMC10765696

[28] Chen TK, Knicely DH, Grams ME. Chronic kidney disease diagnosis and management: a review. JAMA. 2019;322(13):1294–1304. doi: 10.1001/jama.2019.14745. 31573641 PMC7015670

[29] Vavilis G, Bäck M, Occhino G, et al. Kidney dysfunction and the risk of developing aortic stenosis. J Am Coll Cardiol. 2019;73(3):305–314. doi: 10.1016/j.jacc.2018.10.068. 30678761

[30] Guerraty MA, Chai B, Hsu JY, et al. Relation of aortic valve calcium to chronic kidney disease (from the Chronic Renal Insufficiency Cohort Study). Am J Cardiol. 2015;115(9):1281–1286. doi: 10.1016/j.amjcard.2015.02.011. 25791240 PMC4395541

[31] Massera D, Bartz TM, Biggs ML, et al. Traditional and novel risk factors for incident aortic stenosis in community-dwelling older adults. Heart. 2023;110(1):57–64. doi: 10.1136/heartjnl-2023-322709. 37463733 PMC10794538

[32] Samad Z, Sivak JA, Phelan M, et al. Prevalence and outcomes of left-sided valvular heart disease associated with chronic kidney disease. J Am Heart Assoc. 2017;6(10):e006044. doi: 10.1161/JAHA.117.006044. PMC5721834 29021274

[33] Candellier A, Bohbot Y, Pasquet A, et al. Chronic kidney disease is a key risk factor for aortic stenosis progression. Nephrol Dial Transplant. 2023;38(12):2776–2785. doi: 10.1093/ndt/gfad116. 37248048 PMC10689189

[34] Gupta JI, Gualano SK, Bhave N. Aortic stenosis in ­chronic kidney disease: challenges in diagnosis and treatment. Heart. 2022;108(16):1260–1266. doi: 10.1136/heartjnl-2021-319604. 34952860

[35] Disthabanchong S, Srisuwarn P. Mechanisms of vascular calcification in kidney disease. Adv Chronic Kidney Dis. 2019;26(6):417–426. doi: 10.1053/j.ackd.2019.08.014. 31831120

[36] Shroff GR, Bangalore S, Bhave NM, et al. Evaluation and management of aortic stenosis in chronic kidney disease: a scientific statement from the american heart association. Circulation. 2021;143(25):e1088–e1114. doi: 10.1161/CIR.0000000000000979. 33980041

[37] Lerman DA, Prasad S, Alotti N. Denosumab could be a potential inhibitor of valvular interstitial cells calcification in vitro. Int J Cardiovasc Res. 2016;5(1):1 0.4172/2324–8602. doi: 10.4172/2324-8602.1000249. PMC4959558 27468412

[38] Fox CS, Larson MG, Vasan RS, et al. Cross-sectional association of kidney function with valvular and annular calcification: the Framingham heart study. J Am Soc Nephrol. 2006;17(2):521–527. doi: 10.1681/ASN.2005060627. 16382018

[39] Lo R, Narasaki Y, Lei S, et al. Management of traditional risk factors for the development and progression of chronic kidney disease. Clin Kidney J. 2023;16(11):1737–1750. doi: 10.1093/ckj/sfad101. 37915906 PMC10616454

[40] Nazarzadeh M, Pinho-Gomes A-C, Bidel Z, et al. Plasma lipids and risk of aortic valve stenosis: a Mendelian randomization study. Eur Heart J. 2020;41(40):3913–3920. doi: 10.1093/eurheartj/ehaa070. 32076698 PMC7654932

[41] Ciofani JL, Han D, Allahwala UK, et al. Aortic stenosis and renal function: a bidirectional mendelian randomization analysis. J Am Heart Assoc. 2024;13(9):e034102. doi: 10.1161/JAHA.123.034102. 38639330 PMC11179900

[42] Greve AM, Bang CN, Boman K, et al. Effect modifications of lipid-lowering therapy on progression of aortic stenosis (from the simvastatin and ezetimibe in aortic stenosis [SEAS] study). Am J Cardiol. 2018;121(6):739–745. doi: 10.1016/j.amjcard.2017.12.011. 29361285

[43] Raggi P, Bellasi A, Bushinsky D, et al. Slowing progression of cardiovascular calcification with SNF472 in patients on hemodialysis: results of a randomized phase 2b study. Circulation. 2020;141(9):728–739. doi: 10.1161/CIRCULATIONAHA.119.044195. 31707860

