# Supplementary figures and images for: Causal associations between kidney function and aortic valve stenosis: a bidirectional Mendelian randomization analysis
Source: Ren Fail. 2024 Oct 23;46(2):2417742. doi: 10.1080/0886022X.2024.2417742 (PMC11500509; doi:10.1080/0886022X.2024.2417742)

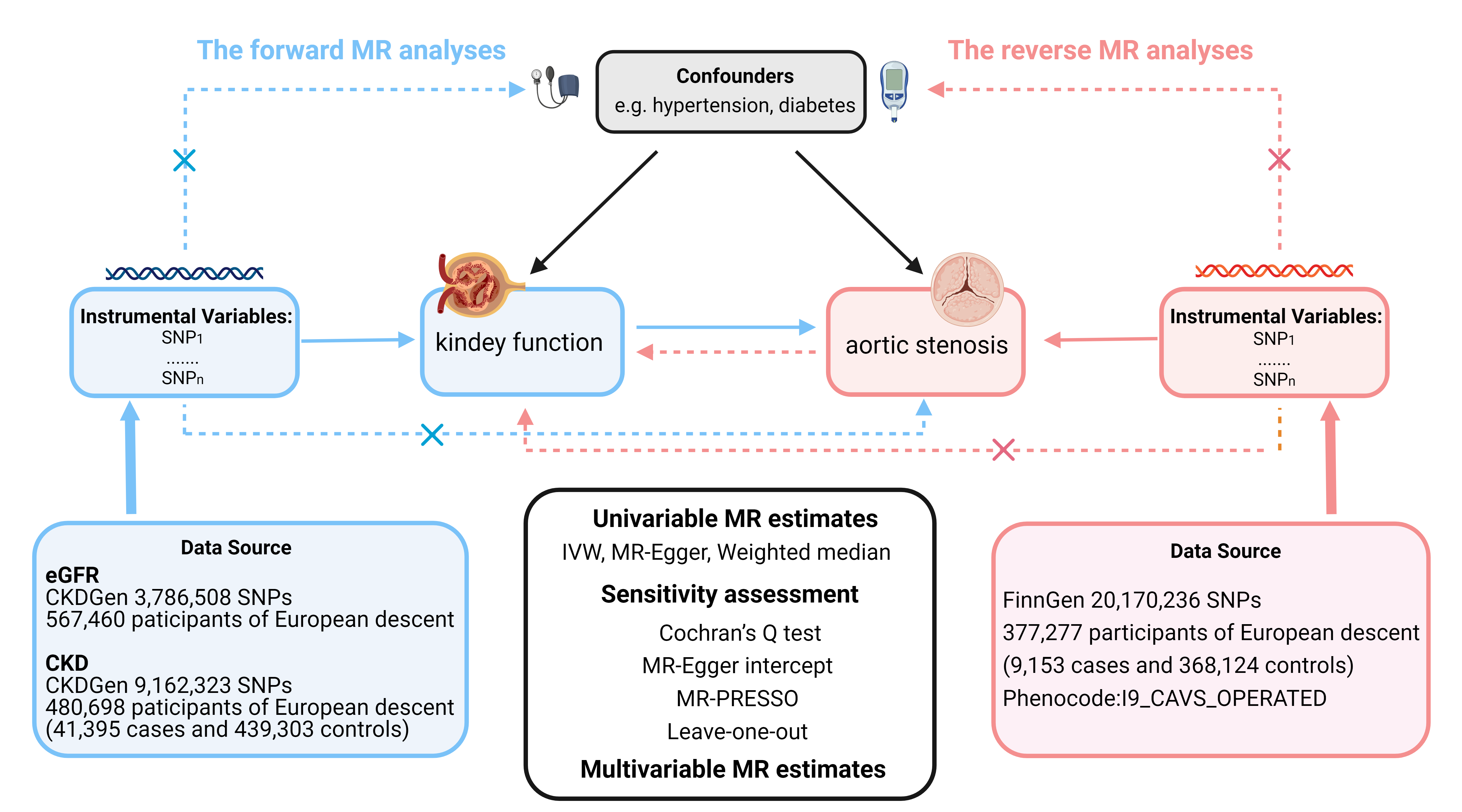

Supplement: Figure1.png [file IRNF_A_2417742_SM4874.png]

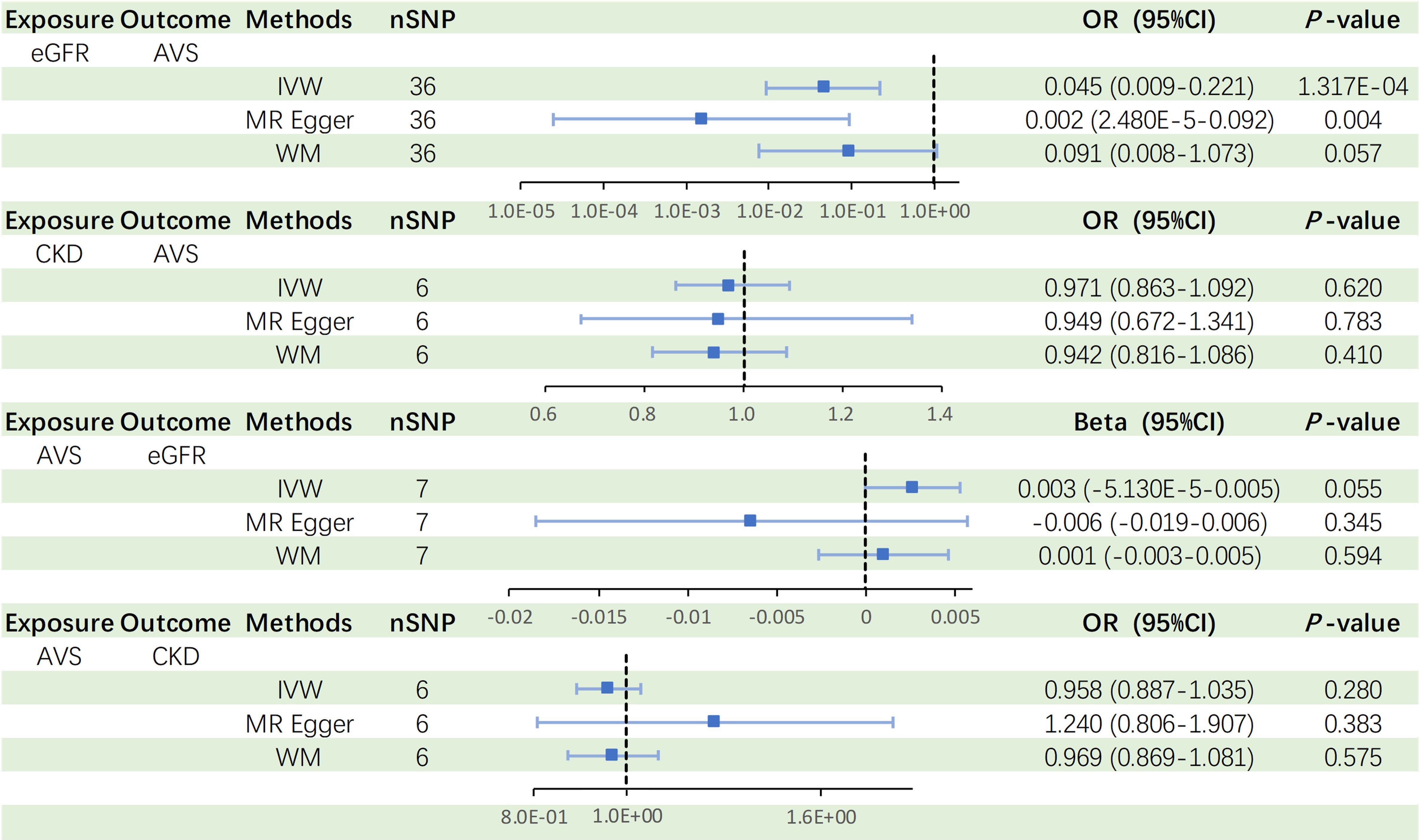

Supplement: Figure2.tif [file IRNF_A_2417742_SM4872.tif]

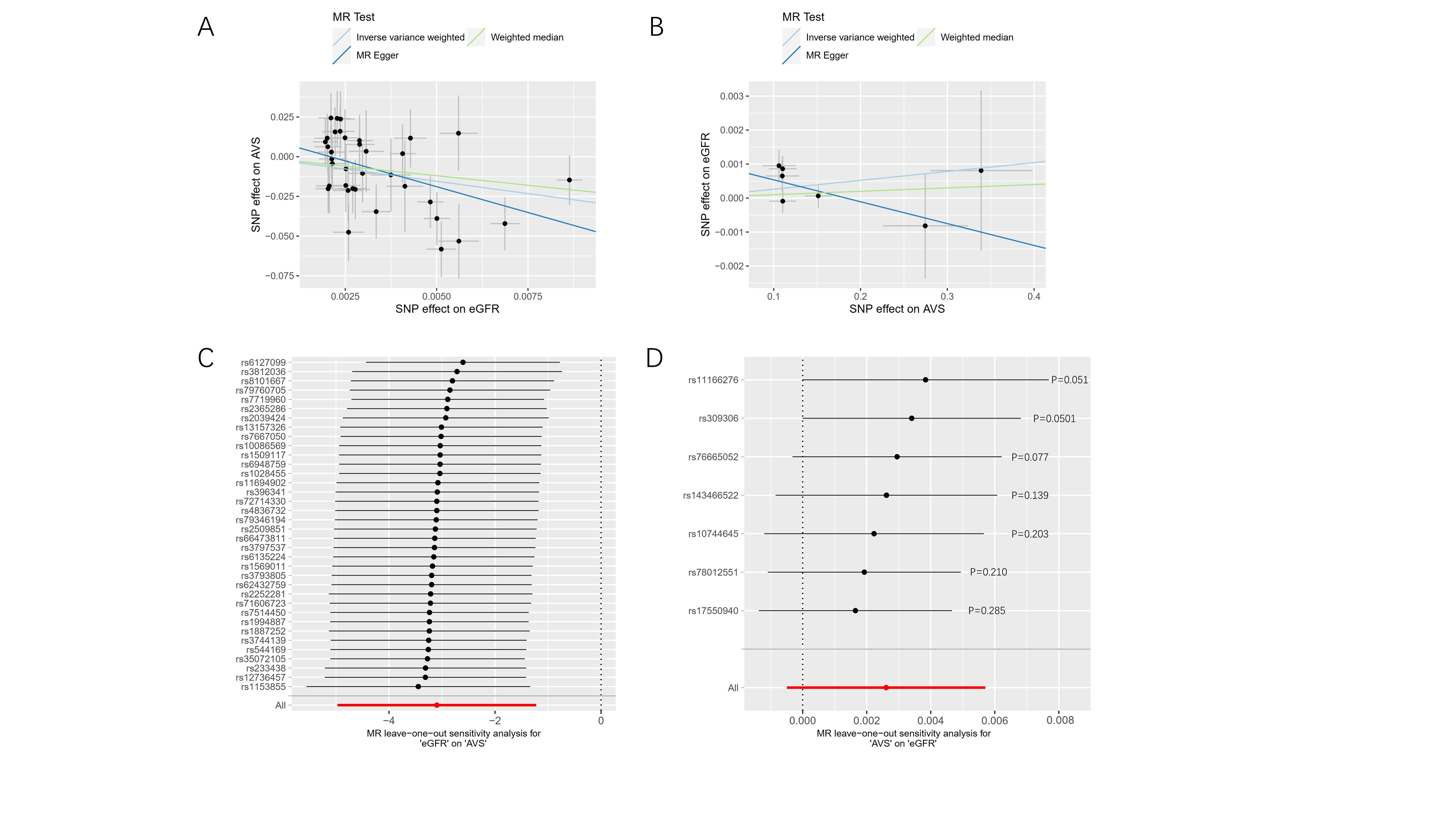

Supplement: Figure3.tif [file IRNF_A_2417742_SM4871.tif]

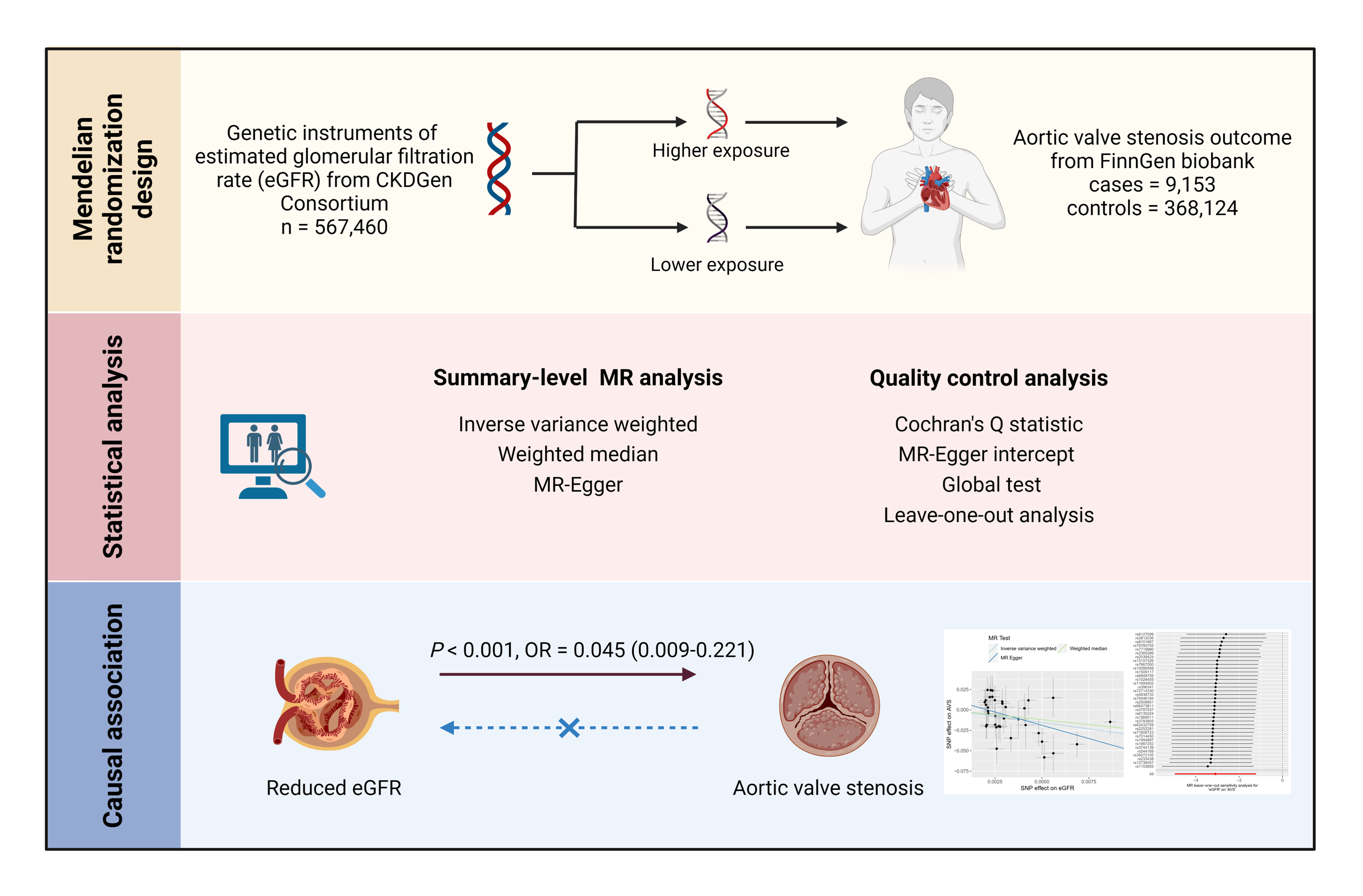

Supplement: Graphical abstract.tif [file IRNF_A_2417742_SM4870.tif]
