# Supplementary material for: Causal associations between kidney function and aortic valve stenosis: a bidirectional Mendelian randomization analysis
Source: Ren Fail. 2024 Oct 23;46(2):2417742. doi: 10.1080/0886022X.2024.2417742 (PMC11500509; doi:10.1080/0886022X.2024.2417742)
Supplement: Supplementary Materials.docx [file IRNF_A_2417742_SM4873.docx]

***Supplementary Materials***

**Causal Associations Between Kidney Function and Aortic Valve Stenosis: A Bidirectional Mendelian Randomization Analysis**

Wanqian Pan, Le Zhou, Rui Han, Xiaojiao Du, Weixiang Chen, Tingbo Jiang

**Contents**

**Table S1.** The detailed information on GWAS data sources.

**Table S2.** Summary statistics of the eGFR genetic instrumental variables.

**Table S3.** Summary statistics of the CKD genetic instrumental variables.

**Table S4.** Summary statistics of the AVS genetic instrumental variables for eGFR reverse MR analysis.

**Table S5.** Summary statistics of the AVS genetic instrumental variables for CKD reverse MR analysis.

**Figure S1.** The forest plots between kidney function and AVS.

**Figure S2.** The scatter plot of three MR analysis methods between AVS and CKD.

**Figure S3.** Predefined decision tree.

**Table S1.** The detailed information on GWAS data sources.

| **Trait** | **Consortium** | **PMID** | **Phenocode** | **Samplesize** | **Population** | **Website link** |
| --- | --- | --- | --- | --- | --- | --- |
| eGFR | CKDGen | 31152163 | \ | 567,460 | European | <https://ckdgen.imbi.uni-freiburg.de/datasets/Wuttke_2019> |
| chronic kidney disease | CKDGen | 31152163 | \ | 480,698 | European | <https://ckdgen.imbi.uni-freiburg.de/datasets/Wuttke_2019> |
| aortic valve stenosis | FinnGen | \ | I9_CAVS_OPERATED | 377,277 | European | <https://www.finngen.fi/en/access_results> |
| body mass index | GIANT | 30124842 | ieu-b-40 | 681,275 | European | <https://gwas.mrcieu.ac.uk/datasets/ieu-b-40/> |
| hypertension | MRC-IEU | \ | ukb-b-12493 | 463,010 | European | https://gwas.mrcieu.ac.uk/datasets/ukb-b-12493/ |
| Type 2 diabetes | \ | 30054458 | ebi-a-GCST006867 | 655,666 | European | <https://gwas.mrcieu.ac.uk/datasets/ebi-a-GCST006867/> |
| LDL-C | \ | 32493714 | ebi-a-GCST90002412 | 431,167 | European | <https://gwas.mrcieu.ac.uk/datasets/ebi-a-GCST90002412/> |
| smoking | MRC-IEU | \ | ukb-b-223 | 462,434 | European | <https://gwas.mrcieu.ac.uk/datasets/ukb-b-223/> |

**Table S2.** Summary statistics of the eGFR genetic instrumental variables.

| SNP | A1 | A2 | Exposure | | | Outcome | | |
| --- | --- | --- | --- | --- | --- | --- | --- | --- |
|  |  |  | Beta | Se | *P*-value | Beta | Se | *P*-value |
| rs1153855 | G | C | 0.008636 | 0.000352 | 1.2E-132 | -0.01469 | 0.015616 | 0.346731 |
| rs12736457 | G | C | 0.0056 | 0.000523 | 8.79E-27 | 0.014758 | 0.023794 | 0.535098 |
| rs1569011 | G | A | 0.001954 | 0.000347 | 1.73E-08 | 0.009298 | 0.015591 | 0.55094 |
| rs2252281 | C | T | 0.004068 | 0.000359 | 8.47E-30 | 0.001912 | 0.018426 | 0.917358 |
| rs3797537 | G | A | 0.00212 | 0.000377 | 1.86E-08 | 0.0029 | 0.017262 | 0.86658 |
| rs396341 | C | T | 0.002973 | 0.000388 | 1.72E-14 | -0.0105 | 0.01827 | 0.565615 |
| rs4836732 | C | T | 0.002518 | 0.000348 | 4.69E-13 | -0.00765 | 0.015619 | 0.624104 |
| rs544169 | G | A | 0.00237 | 0.000388 | 9.98E-10 | 0.023676 | 0.017396 | 0.173512 |
| rs6127099 | T | A | -0.00513 | 0.000406 | 1.17E-36 | 0.058195 | 0.017514 | 0.000891 |
| rs10086569 | C | T | 0.002774 | 0.000403 | 5.73E-12 | -0.02061 | 0.018694 | 0.27021 |
| rs1028455 | T | A | 0.002062 | 0.000367 | 1.90E-08 | -0.01843 | 0.017042 | 0.279484 |
| rs11694902 | G | A | 0.004132 | 0.000503 | 2.14E-16 | -0.01862 | 0.02882 | 0.518159 |
| rs13157326 | G | A | -0.00271 | 0.000388 | 2.95E-12 | 0.020068 | 0.015629 | 0.199157 |
| rs1509117 | T | A | 0.002513 | 4.00E-04 | 3.33E-10 | -0.01814 | 0.016681 | 0.276957 |
| rs1887252 | G | C | -0.00289 | 0.000358 | 7.45E-16 | -0.01013 | 0.016117 | 0.529701 |
| rs1994887 | C | A | -0.00236 | 0.000395 | 2.34E-09 | -0.01596 | 0.0161 | 0.32154 |
| rs2039424 | G | A | 0.004828 | 0.000361 | 9.75E-41 | -0.0285 | 0.016471 | 0.083526 |
| rs233438 | G | A | 0.004284 | 0.000441 | 2.84E-22 | 0.011678 | 0.018411 | 0.525909 |
| rs2365286 | G | A | -0.00335 | 0.000394 | 2.08E-17 | 0.0346 | 0.016987 | 0.04167 |
| rs2509851 | C | A | 0.002132 | 0.000354 | 1.62E-09 | -0.00142 | 0.016054 | 0.92933 |
| rs35072105 | G | A | -0.00211 | 0.000351 | 1.97E-09 | -0.02438 | 0.01566 | 0.119471 |
| rs3744139 | G | T | 0.00228 | 0.000363 | 3.39E-10 | 0.024116 | 0.017439 | 0.16671 |
| rs3793805 | G | A | -0.00201 | 0.000351 | 1.03E-08 | -0.01167 | 0.015519 | 0.452211 |
| rs3812036 | C | T | -0.00687 | 0.000406 | 3.19E-64 | 0.042142 | 0.01668 | 0.011523 |
| rs6135224 | G | A | -0.00202 | 0.000369 | 4.04E-08 | -0.00625 | 0.017008 | 0.713145 |
| rs62432759 | G | A | -0.00249 | 0.000432 | 7.56E-09 | -0.01186 | 0.018086 | 0.511835 |
| rs66473811 | C | T | 0.003072 | 0.000483 | 2.02E-10 | 0.003374 | 0.025846 | 0.896151 |
| rs6948759 | C | T | -0.00258 | 0.000422 | 1.01E-09 | 0.021271 | 0.019406 | 0.273042 |
| rs71606723 | T | A | 0.002893 | 0.000405 | 9.26E-13 | 0.007701 | 0.016323 | 0.637087 |
| rs72714330 | C | T | 0.003749 | 0.00055 | 9.20E-12 | -0.01148 | 0.02295 | 0.617021 |
| rs7514450 | C | T | 0.002222 | 0.000347 | 1.48E-10 | 0.015601 | 0.015459 | 0.312874 |
| rs7667050 | C | T | 0.002031 | 0.000342 | 2.98E-09 | -0.0202 | 0.015369 | 0.188742 |
| rs7719960 | G | A | 0.00259 | 0.000417 | 5.24E-10 | -0.04755 | 0.018235 | 0.009112 |
| rs79346194 | G | A | -0.00215 | 0.000377 | 1.22E-08 | 0.004403 | 0.016367 | 0.787913 |
| rs79760705 | G | T | 0.005609 | 0.000551 | 2.55E-24 | -0.05316 | 0.023578 | 0.024148 |
| rs8101667 | C | T | 0.005007 | 0.000363 | 2.19E-43 | -0.03893 | 0.016706 | 0.019792 |

**Table S3.** Summary statistics of the CKD genetic instrumental variables.

| SNP | A1 | A2 | Exposure | | | Outcome | | |
| --- | --- | --- | --- | --- | --- | --- | --- | --- |
|  |  |  | Beta | Se | *P*-value | Beta | Se | *P*-value |
| rs700221 | G | A | -0.0719 | 0.0098 | 2.19E-13 | 0.010944 | 0.015711 | 0.486086 |
| rs35716097 | C | T | 0.0785 | 0.0105 | 8.20E-14 | 0.031428 | 0.015938 | 0.048625 |
| rs1049518 | G | A | 0.0788 | 0.0094 | 5.42E-17 | -0.0148 | 0.015614 | 0.343226 |
| rs3925584 | C | T | 0.08 | 0.0092 | 4.68E-18 | -0.01561 | 0.015602 | 0.31719 |
| rs10224002 | G | A | -0.1083 | 0.0102 | 2.65E-26 | -0.00809 | 0.018215 | 0.656789 |
| rs77924615 | G | A | -0.2237 | 0.0128 | 6.38E-69 | 0.011574 | 0.018577 | 0.533267 |

**Table S4.** Summary statistics of the AVS genetic instrumental variables for eGFR reverse MR analysis.

| SNP | A1 | A2 | Exposure | | | Outcome | | |
| --- | --- | --- | --- | --- | --- | --- | --- | --- |
|  |  |  | Beta | Se | *P*-value | Beta | Se | *P*-value |
| rs10744645 | C | T | 0.109837 | 0.019413 | 1.53E-08 | 0.00065 | 0.000466 | 0.1625 |
| rs11166276 | T | C | 0.151239 | 0.015367 | 7.43E-23 | 6.50E-05 | 0.000341 | 0.8484 |
| rs143466522 | A | G | 0.338965 | 0.058883 | 8.58E-09 | 0.00081 | 0.00235 | 0.7304 |
| rs17550940 | C | A | 0.110264 | 0.015754 | 2.58E-12 | 0.000862 | 0.000379 | 0.02306 |
| rs309306 | T | C | -0.11036 | 0.015538 | 1.22E-12 | 9.20E-05 | 0.000345 | 0.7907 |
| rs76665052 | C | A | -0.27455 | 0.048917 | 1.99E-08 | 0.000818 | 0.001551 | 0.5979 |
| rs78012551 | G | A | 0.106054 | 0.019202 | 3.33E-08 | 0.000952 | 0.000463 | 0.0396 |

**Table S5.** Summary statistics of the AVS genetic instrumental variables for CKD reverse MR analysis.

| SNP | A1 | A2 | Exposure | | | Outcome | | |
| --- | --- | --- | --- | --- | --- | --- | --- | --- |
|  |  |  | Beta | Se | *P*-value | Beta | Se | *P*-value |
| rs10744645 | C | T | 0.109837 | 0.019413 | 1.53E-08 | -0.0307 | 0.0124 | 0.01356 |
| rs11166276 | T | C | 0.151239 | 0.015367 | 7.43E-23 | 0.0097 | 0.0091 | 0.286 |
| rs143466522 | A | G | 0.338965 | 0.058883 | 8.58E-09 | -0.0903 | 0.0726 | 0.2135 |
| rs309306 | T | C | -0.11036 | 0.015538 | 1.22E-12 | 0.0103 | 0.0091 | 0.2575 |
| rs76665052 | C | A | -0.27455 | 0.048917 | 1.99E-08 | -0.0216 | 0.0389 | 0.5779 |
| rs78012551 | G | A | 0.106054 | 0.019202 | 3.33E-08 | -0.0111 | 0.0124 | 0.3718 |

**
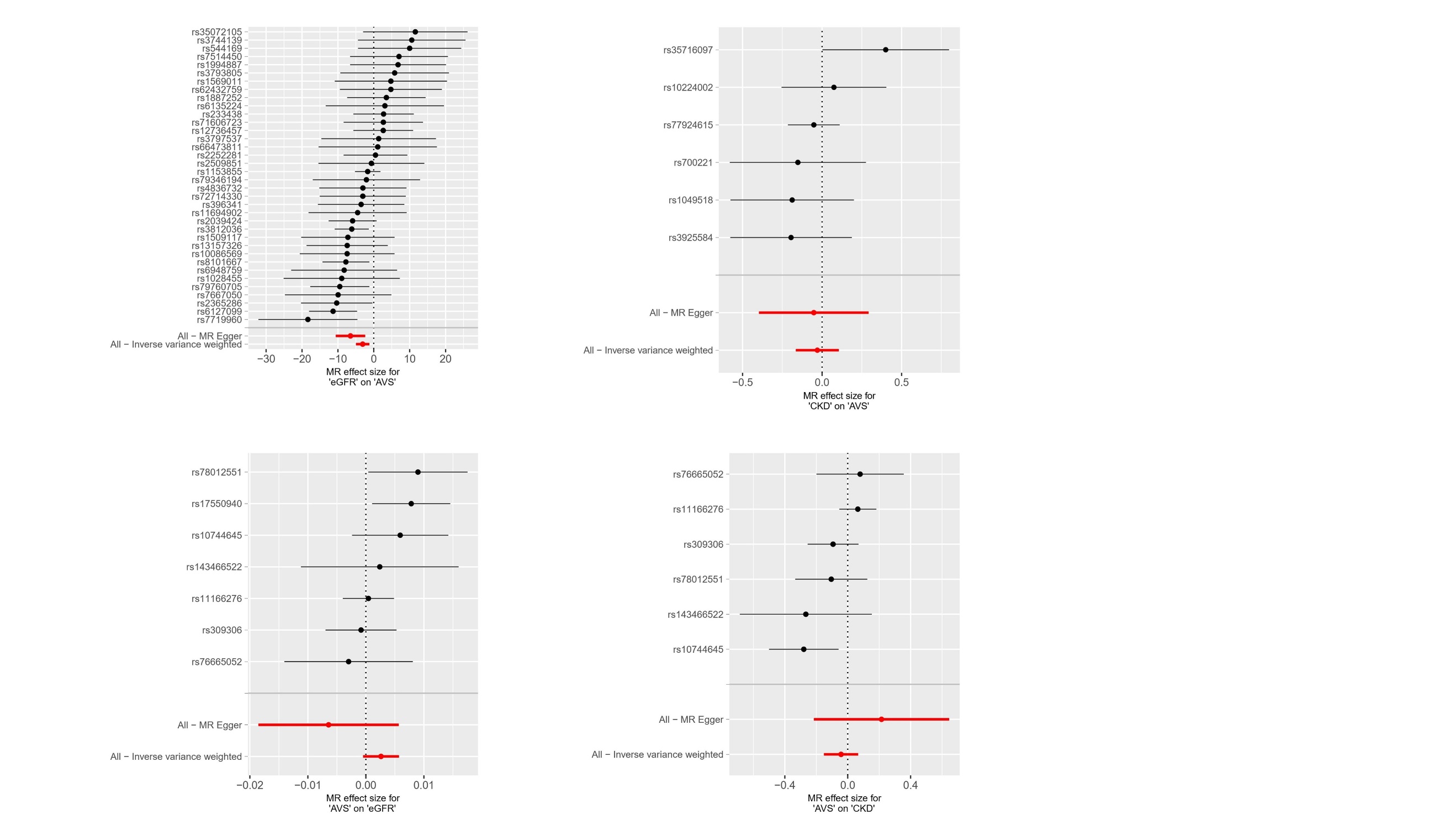
**

**Figure S1.** The forest plots between kidney function and AVS.


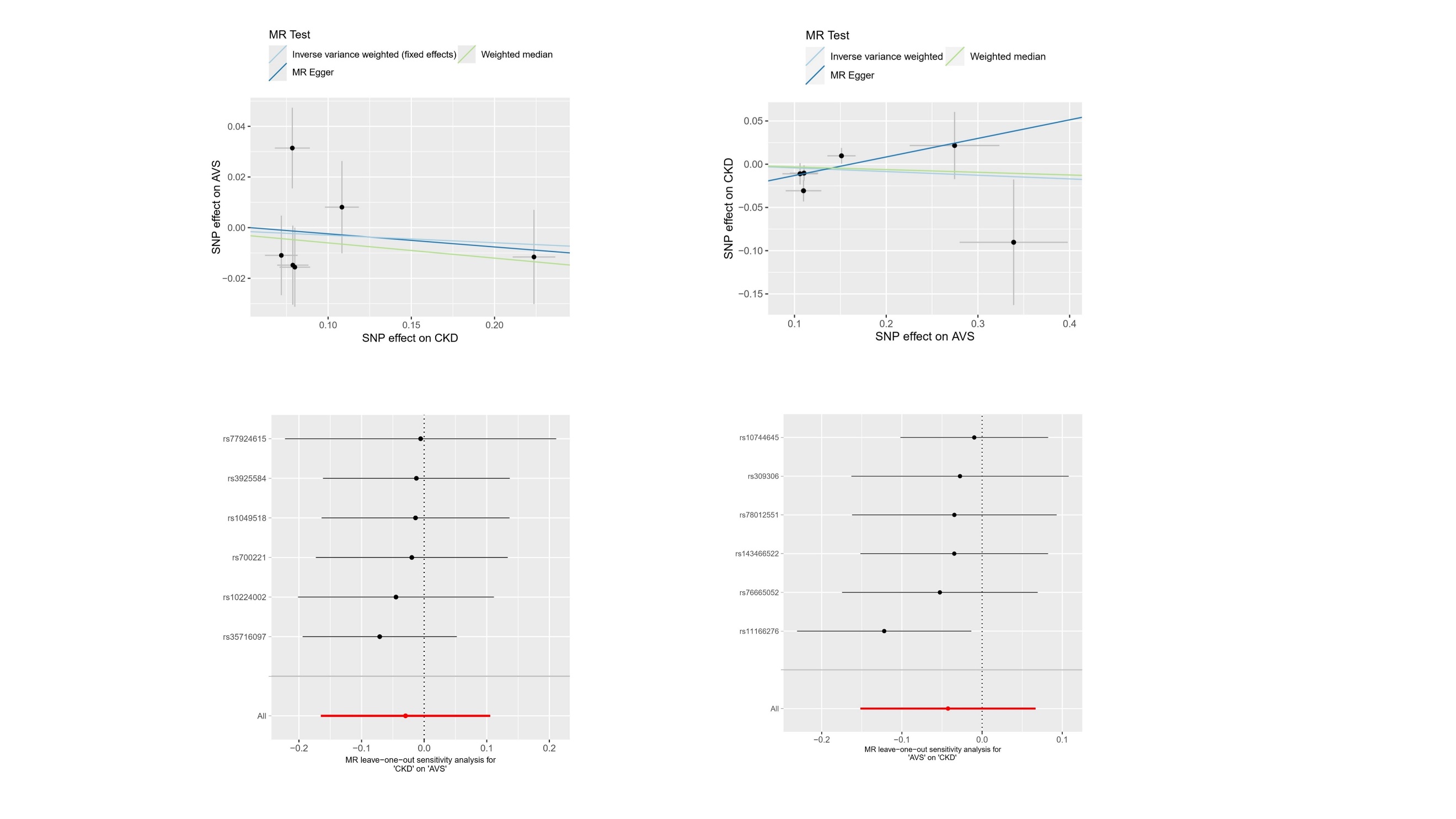


**Figure S2.** The scatter plot of three MR analysis methods between AVS and CKD.


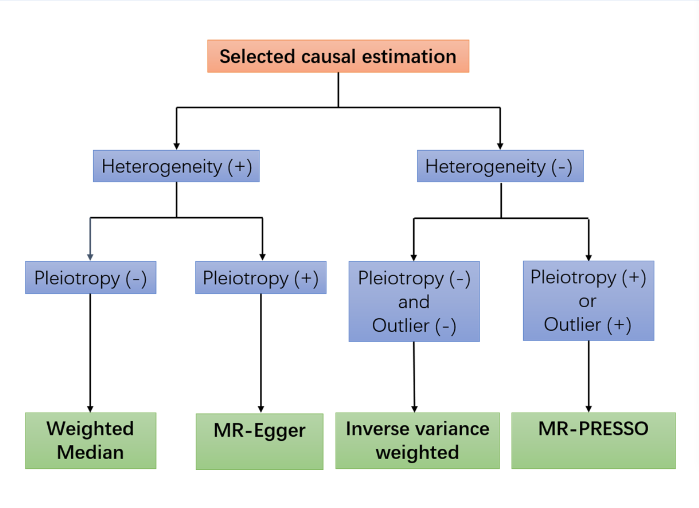


**Figure S3.** Predefined decision tree.
